# Evaluation of tumorigenesis-related miRNAs in breast cancer in Egyptian women: a retrospective, exploratory analysis

**DOI:** 10.1038/s41598-024-68758-0

**Published:** 2024-11-29

**Authors:** Ghada M. Salum, Nesma M. Elaraby, Hoda A. Ahmed, Mai Abd El Meguid, Basma E. Fotouh, Muhammed Ashraf, Yasmine Elhusseny, Reham M. Dawood

**Affiliations:** 1 Department of Microbial Biotechnology, Biotechnology Research Institute, National Research Centre, EL Bohouth St. (Former El Tahrir St.), Dokki, P.O. 12622, Giza, Egypt; 2 Medical Molecular Genetic Department, Human Genetics and Genome Research Institute, National Research Centre, P.O. 12622, Dokki, Giza, Egypt; 3 Egypt Centre for Research and Regenerative Medicine, ECRRM, Cairo, 11517 Egypt; 4 Medical Biochemistry and Molecular Biology Department, School of Medicine, NewGiza University, Giza, Egypt

**Keywords:** Biochemistry, Biotechnology

## Abstract

Breast cancer (BC) is a leading cause of global female cancer-related deaths, despite treatment advancements. A growing focus on investigating microRNA-based therapeutics and their role in BC progression. A computational analysis was performed to identify the potential miRNA–mRNA network involved in the BC pathogenesis and assist with the treatment strategy. Then, the expression levels of five circulatory miRNAs (miR-200a-3p, miR-124-3p, miR-205-5p, miR-15a-5p, and miR-155-5p) were assessed by using qRT-PCR in 75 BC patients (early-stage: n = 26 and late-stage: n = 49) and 20 healthy controls. The analysis included various (a) stages (early and late) and (b) receptor statuses (ER + ve & HER2 -ve), (HER + ve & ER -ve), and triple-negative (TNBC). In-silico analysis suggested that *STAT3* serves as an efficacy biomarker suppressed by miR-124-3p. Additionally, the miR-155-5p showed the ability to activate *CTNNB1* which acts as a biomarker for BC progression, to inhibit DNA repair genes (*ARID2*, and *WEE1*)*,* and the transcriptional factor gene (*TCF4*). MiR-205-5p and miR-16 suppressed *VEGFA* expression, a survival factor for BC. MiR-200a-3p, miR-205-5p, and miR-124-3p showed downregulation in the serum of BC patients compared to controls. The ROC analysis of those miRNAs demonstrated their significant diagnostic accuracy for identifying BC patients. Additionally, miR-155-5p exhibited a significant upregulation in TNBC and can be used as an indicative marker for TNBC. This study holds significant promise for the development of noninvasive miRNA biomarkers with potential clinical applications.

## Introduction

Breast cancer (BC) is a leading cause of global female cancer-related deaths despite treatment advancements^1–3^. In Egypt, BC is the most common type of cancer that affects women. It is estimated that in the year 2050, there will be around 46,000 new infected cases^4^. BC is classified into five molecular subtypes: luminal A, luminal B, luminal B, and human epidermal growth factor receptor 2 (HER2) positive, HER2-enriched, and triple-negative (TNBC). A better prognosis is associated with luminal A& B subtypes. Luminal A is the most prevalent subtype (50–60% of cases); it is identified by the expression of BCl-2, progesterone receptor (PR), estrogen receptor (ER), and the lack of HER2. The absence of HER2 and the presence of PR and ER identify the luminal B subtype^5^. Around 80% of BC cases express ER. In contrast, approximately 15% of BC cases exhibit overexpression of HER2. The latter subset of BC is characterized by a heightened rate of proliferation which makes understanding its molecular characteristics a must be^2,6^. The remaining percentage of BC (5%), known as TNBC, has a poor prognosis and does not express any of the three markers (ER, PR, and HER2)^7^. BC, a complex and diverse disease, requires an understanding of its molecular and clinical characteristics. Fine Needle Aspiration Cytology (FNAC) is a less invasive diagnostic procedure for only small breast lesions and is often guided by ultrasound or mammography^8^. FNAC may not always yield enough tissue for a definitive diagnosis, so, a core biopsy may be performed for screening the tumor architecture as it is more invasive than FNAC^9^. The transcriptomes are increasingly validated as powerful biomarkers in many cancers^10–12^, including BC^13^. These biomarkers are expected to overcome mammography's limitations when utilized as a complementary diagnostic tool^12^. One of the transcriptome sets is the micro RNAs (miRNAs; small non-coding RNAs) which widely vary across many tissue types and different cancers^5^. The miRNA profiling approach indicated the involvement of miRNA dysregulation in BC. Furthermore, it is known that the circulating miRNA levels return to baseline following tumor resection. This finding supports the hypothesis that circulating miRNAs could be useful biomarkers for prognosis prediction in early-stage breast cancer and for evaluating the efficacy of cancer therapies^14^. Remarkably, it has been demonstrated that some miRNAs can cause oncogenesis such as miR-155-5p while other miRNAs known as tumor suppressor miRNAs—work as inhibitors of tumor growth such as miR-200a-3p, miR-205-5p, miR-124-3p, and miR-15a-5p^15^.

It was reported that miR-155-5p is a prime regulator in inducing epithelial-mesenchymal transition (EMT); the state at which the carcinoma cells lose epithelial characteristics and acquire cell motility to achieve invasion^12^. The miR-200a-3p, a part of the miR-200 family with members like miR-200b, miR-200c, miR-141, and miR-429, controls EMT by targeting transcription factors like *TGFβ2*, *ZEB1*, and *ZEB2*. Several evidence indicated that the overexpression of miR200a inhibits TNBC cell death and thereby enhances chemotherapy resistance^16,17^.

Further investigation on the mechanical functions of these miRNAs and how they contribute to the disease progression may improve the diagnostic methods and develop specialized therapeutic strategies. Based on the previous study, the current research focused on five miRNAs that had a potential role in liver cancer^18^. The Insilco analysis has been performed to predict the implication and the interaction of the selected miRNA- with the BC-related genes. Then, by using qRT-PCR, we aimed to examine the levels of expression for five miRNAs in the serum of healthy individuals as well as BC patients, including (a) different stages; early and late stages, and (b) three diverse receptor statuses; ER positivity (ER + ve& HER-2 -ve), HER2 positivity (HER + ve & ER -ve), and the TNBC subtype.

## Results

### Pathological parameters of BC patients

In this study, seventy-five breast cancer patients with different stages (early 0, I, II; n = 26 and late III, IV; n = 49) were enrolled. The age of all subjects was between 18 and ≤ 75 years. Based on molecular subtyping, most patients in both groups included IDC-BIRADS 4c/5/6 with 91% of late-stage cases and 87% of early-stage cases. Lymph node involvement showed the presence of N1 in late-stage cases (38%) and N0 in early-stage cases (54%). Tumor size is classified into T1, T2, T3, and T4. Early-stage patients have a higher percentage of T2 tumors (58%), while the higher percentage of late-stage patients is T4 tumors (54%). Both late-stage and early-stage patients have the pre and post-menopausal status. The post-menopausal group represents about 56% of early and 46% of late stage. Table 1 displays all the pathological parameters of all patients.

**Table 1:** Patient Demographics and Characteristics.

Parameters	Patients
	N = 75
Age	< 45 33%
	> 45 67%
Molecular Subtype	IDC-BIRADS 4c/5/6 86%
	Benign Phyllodes Tumor 1%
	Mixed 4c/5/6 13%
ER	Negative 21%
	Positive 79%
PR	Negative 31%
	Positive 68%
HER 2	Negative 72%
	Positive 28%
Grade	G1 3%
	G2 71%
	G3 25%
N	N0 34%
	N1 41%
	N2 12%
	N3 13%
Stage	0 1%
	I 5%
	II 38%
	III 56%
	IV 1%
Laterality	bilateral 4%
	LEFT 48%
	Right 48%
Menopausal status	PRE 45%
	POST 54%
	history of hysterectomy 1%

### In silico analysis for intricate interactions and potential biomarkers in miRNA-mediated regulation of BC pathways

**Figure 1:**
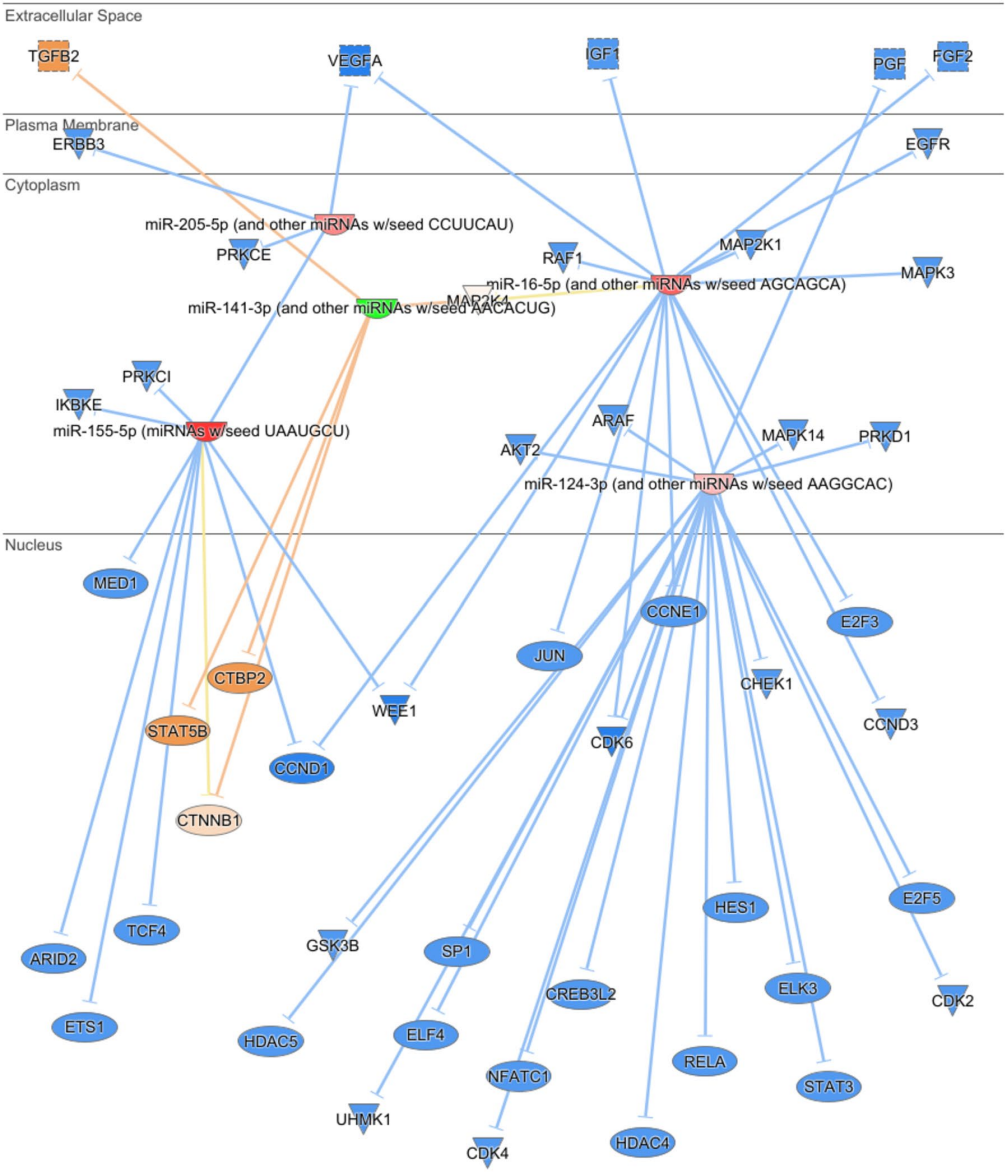
A Simulated Analysis Depicting the Predicted Target Genes and Their Subcellular Localizations in Breast Cancer. The figure was generated utilizing Ingenuity Pathway Analysis (IPA). Triangles represent kinases, oval shapes denote transcriptional regulators, and incomplete oval shapes indicate mature miRNA (the red color indicates an increased measurement, orange oval shapes signify predicted activation, blue one indicates predicted inhibition and green oval shapes represent decreased measurements). Predicted relationships (orange lines indicate activation, blue lines indicate inhibition and yellow lines indicate inconsistency with a state of the downstream molecule). It is noteworthy that dark colors indicate extremes and faint colors indicate less pronounced effects.

After simulating the analysis of thousands of interactions between miRNAs and their mRNA targets to understand the potential mechanisms of action of these miRNAs, a total of 48 mRNAs were found to interact with five selected miRNAs causing inhibition in most cases (Fig. 1). Despite miR-141 and miR-200a-3p having distinct functions, they belong to the miR-200 family and share the same nomenclature in the IPA database^23^. On the other hand, miR-16a and miR-15a-5p, with different functions, also share the same nomenclature in the IPA database (see supplementary Figure S1).

Most interactions occurred with nuclear proteins (30 genes) and some cytoplasmic ones (11 genes). Highlighting the significance of interactions with extracellular growth factors such as *VEGFA* and *IGF1* in cancer growth and progression, several targets, including *CCND1*, *WEE1*, *VEGFA*, and *CDK6*, were identified to interact with two miRNAs. Both miR-155-5p and miR-16-5p, inhibited the same mRNA targets, namely *CCND1* and *WEE1*. The analysis showed the ability of miR-124-3p to interact and inhibit 21 mRNA genes, while miR-205-5p could inhibit only 4 mRNA genes (Fig. 1).

**Figure 2:**
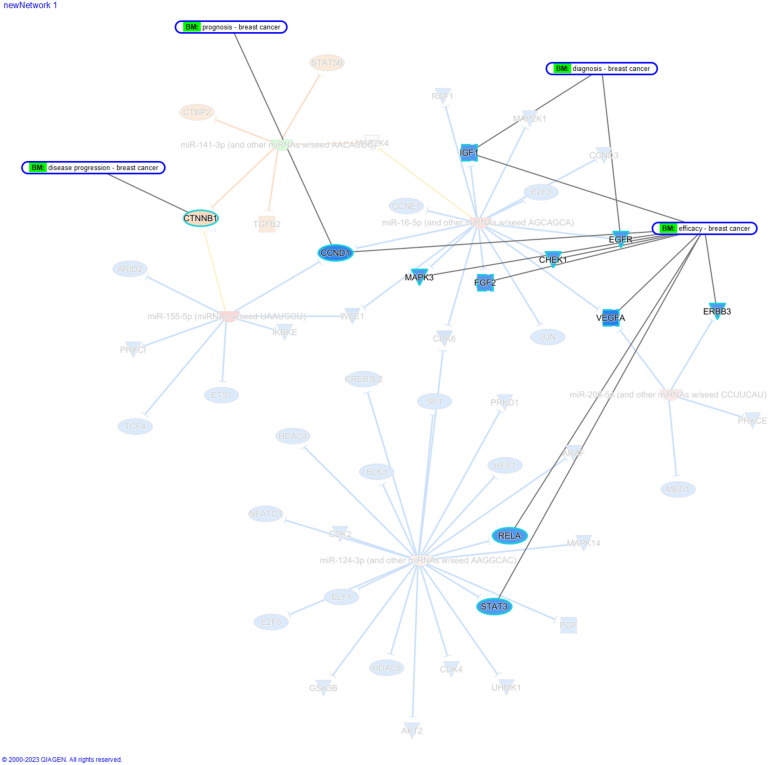
A computational analysis of biomarkers in breast cancer. The figure was generated using IPA (Ingenuity Pathway Analysis).

The potential ability of the studied miRNAs to interact with biomarker genes has been illustrated in (Fig. 2). It was observed that several of the interacted genes are suitable for biomarker applications. The analysis revealed that Cyclin D1 (CCND1) can serve as a potential prognostic and efficacy biomarker.

While Catenin Beta 1 (*CTNNB1*) is the only disease progression biomarker. Analysis has identified seven efficacy biomarkers, such as Vascular Endothelial Growth Factor A (*VEGF-A*), Cyclin D1 (*CCND1*), Receptor Tyrosine Kinase ERBB3 (*ERBB3*), Fibroblast Growth Factor 2 (*FGF2*), Transcription Factor RELA (*RELA*), Signal Transducer and Activator of Transcription 3 (*STAT3*), and Mitogen-Activated Protein Kinase 3 (*MAPK3*). Also, Insulin-like Growth Factor 1 (*IGF1*), and Epidermal Growth Factor Receptor (*EGFR*) have potential diagnostic and efficacy biomarkers.

### Differential expression analysis of the studied miRNAs between breast *cancer* patients and controls

**Figure 3:**
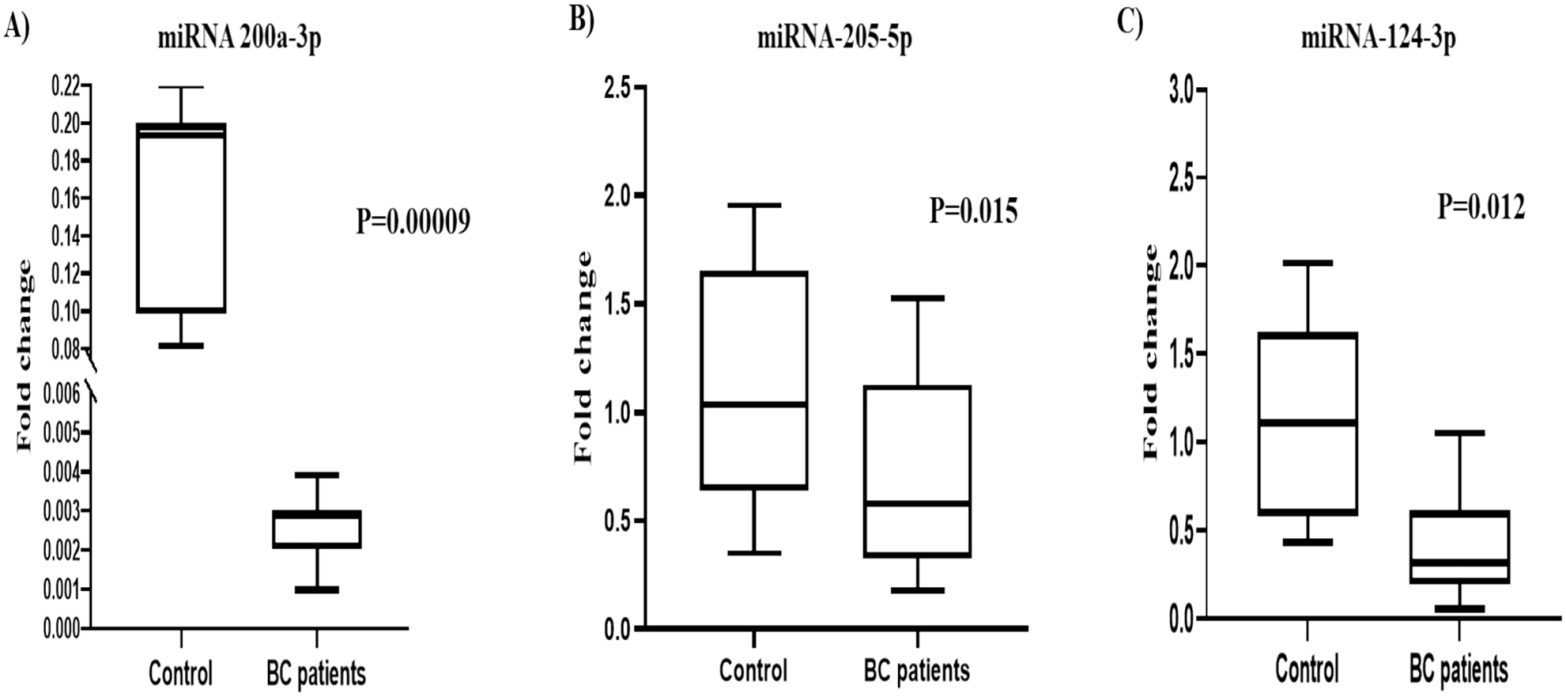
Diagrammatic representation illustrated the expression levels of miRNAs (**A**) miR-200 a, (**B**) miR-205-5p, and **(C)** miR-124-3p in the serum of BC patients (n = 75) versus healthy controls (n = 20). QRT-PCR evaluated the expression levels of the miRNAs. The presented data are depicted as the fold-change relative to the mean of healthy controls, following normalization to the expression of SNORD61.

The study analyzed the serum expression levels of 5 miRNAs in 75 BC patients and 20 healthy controls as illustrated in Table 2. The miRNA expression levels were normalized using the mean of the HK (SNORD 61) gene. The following three miRNAs; miR-200a-3p, miR-205-5p, and miR-124-3p showed significant dysregulation between the control group and patients (*p* = 0.00009, 0.02, 0.01; respectively as depicted in Fig. 3A, B, and C, suggesting their potential role as tumor suppressors.

**Table 2:** Differential expression analysis of the studied miRNAs between breast cancer patients and controls and their implications for tumor suppression and oncogenic activity.

miRNA	Function	Control N = 20	BC Patients N = 75	*P* value
**miR-200a-3p**	**Tumor Suppressor 19**	**0.19(0.1–0.2)**	**0.003(0.002–0.003)**	**0.00009**
**miR-205-5p**	**Tumor Suppressor 20**	**1.04(0.6–1.6)**	**0.5(0.2–1.1)**	**0.015**
**miR-124-3p**	**Tumor Suppressors21**	**1.1 (0.6–1.7)**	**0.25(0.12–0.57)**	**0.012**
**miR-15a-5p**	**Tumor Suppressors 20**	**0.9(0.3–1.3)**	**1.3(0.3–1.9)**	0.2
**miR-155-5p**	**Oncogenic 22**	**1.07(0.4–2.1)**	**0.92(0.22–3.32)**	0.72

Data are expressed as median and interquartile range (IQR).

Bold indicated significant *P* value.

**Figure 4:**
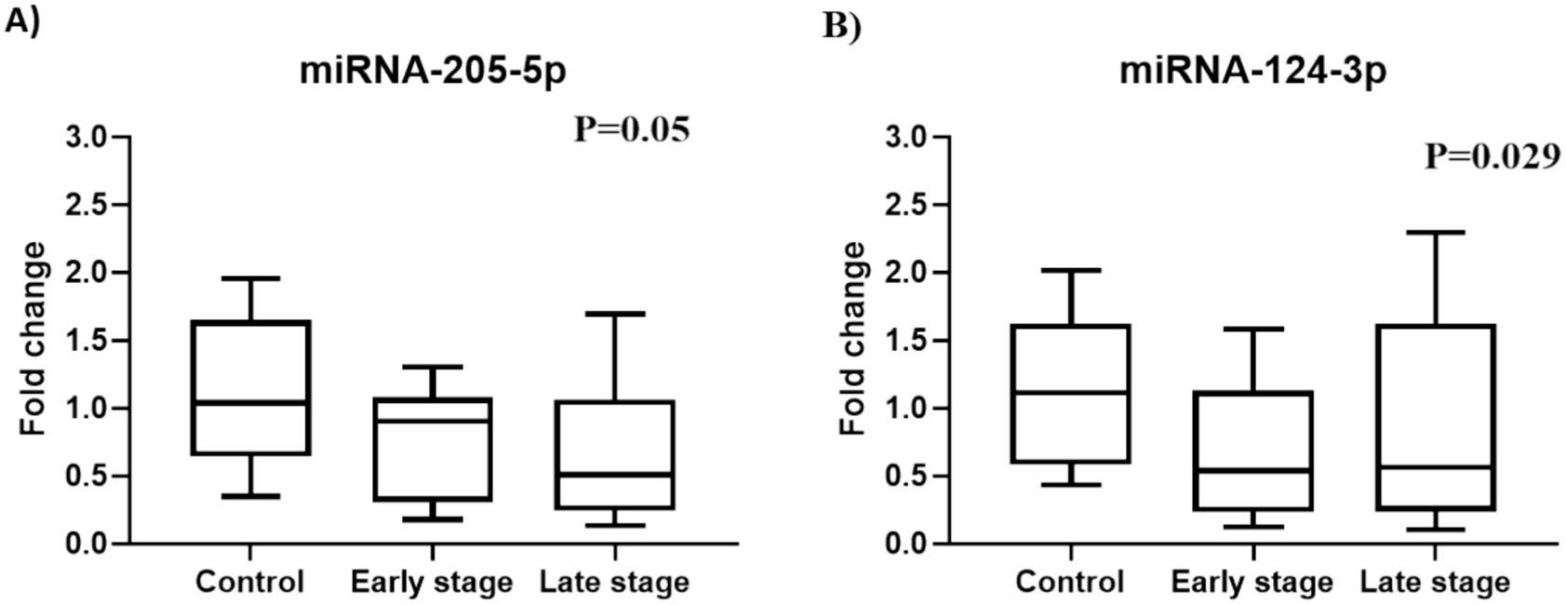
Diagrammatic representation illustrates the expression levels of miRNAs (**A**) miR-205-5p, and (**B**) miR-124-3p in the serum of breast cancer patients at different stages (early 0, I, II; n = 26 and late III, IV; n = 49) and healthy controls (n = 20). The qRT-PCR evaluated the expression levels of miRNAs. The presented data are depicted as the fold-change relative to the mean of healthy controls, following normalization to the expression of SNORD61.

Further analysis has been performed to evaluate the role of five miRNAs on the disease progression. Based on disease progression, patients are categorized into 2 groups early-stage (n = 26), and late-stage (n = 49) compared to the control group (n = 20) as shown in Table 3. The data revealed a downregulation of miR-200a-3p in the early-stage group, and further decline was observed in the late-stage group compared to the control group without significant changes (*P* = 0.24). Similarly, both miR-205-5p and miR-124-3p displayed significant downregulation between the early and late-stage group, and the control group (*P* = 0.05, *P* = 0.02; respectively) as shown in Fig. 4(**A and B**).

**Table 3:** Differential Expression Analysis of the studied miRNAs Across Breast Cancer Stages (Late, Early) and Controls.

miRNA	Control N = 20	Early stageN = 26	Late stageN = 49	*P* value
miR-200a-3p	0.19(0.1–0.2)	0.02(0.006–0.04)	0.003(0.001–0.02)	0.24
miR-205-5p	1.04(0.6–1.6)	0.8(0.3–0.99)	0.5 (0.2–1.1)	**0.05**
miR-124-3p	0.5 (0.2–1.6)	0.5 (0.2–1.1)	1.1 (0.6–1.66)	**0.029**
miR-15a-5p	0.9 (0.3–1.3)	1.4 (0.5–2.8)	1.3 (0.4–3.2)	0.6
miR-155-5p	1.07 (0.4–2.1)	0.6 (0.23–1.9)	1.5 (0.5–3.7)	0.2

Data are expressed as median and interquartile range (IQR).

Bold indicated significant *P* value.

In contrast, the expression levels of miR-155-5p, and miR-15a-5p were upregulated in late-stage breast cancer patients compared to early-stage patients as well as the control group; albeit without statistical significance (*P* = 0.2, P = 0.6; respectively), highlighting potential trends that warrant further investigation.

### Differential expression analysis of the studied miRNAs between breast *cancer* patients and controls based on receptor status

**Figure 5:**
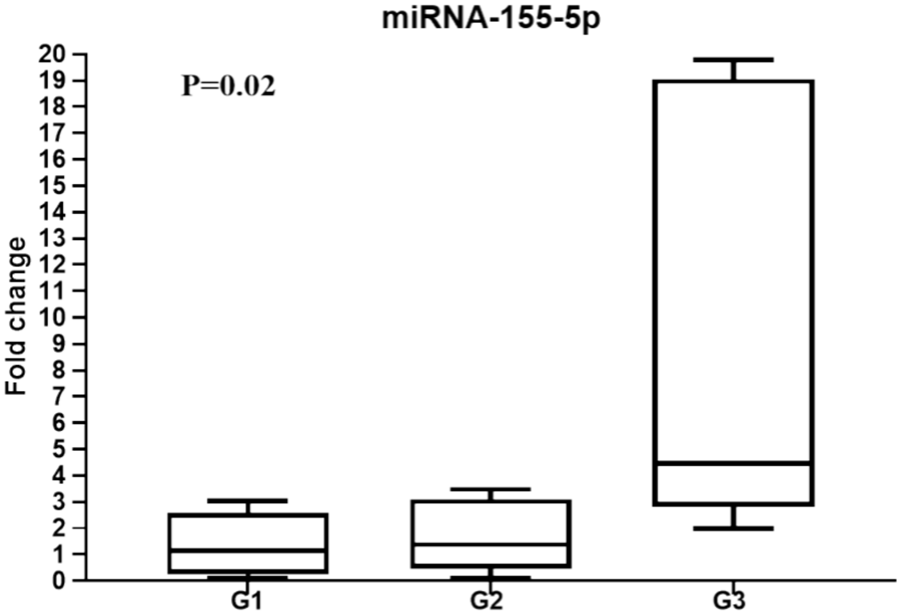
Diagrammatic representation illustrates miR-155-5p expression level in breast cancer patients with different receptor statuses; G1; ER positivity (ER + ve & HER2  - ve), G2; HER2 positivity (HER + ve & ER −ve), and G3; TNBC subtype. qRT-PCR was used to evaluate miR-155-5p expression level and the data are depicted as the fold-change relative to the mean of healthy controls, following normalization to the expression of SNORD61.

To evaluate the effect of the expression level of the studied miRNAs with the clinical pathological parameters, particularly the hormonal status, the enrolled patients were classified into three diverse receptor statuses; G1; ER positivity (ER + ve & HER2 -ve), G2; HER2 positivity (HER + ve & ER -ve), and G3; TNBC subtype as illustrated in Table 4. The data showed that the expression level of miR-200a-3p was upregulated in G2 compared to G1, while its expression level was undetectable in G3, without significant difference. While, the miR-205-5p demonstrated gradual decrement across all groups G1, G2, and G3; *p* = 0.8). Additionally, the downregulation of miR-124-3p and the upregulation of the miR-15a-5p were observed in G3 compared to G1 and G2 with no significant difference (*p* = 0.7, *p* = 0.2). on the other hand, the miR-155-5p displayed the highest expression in G3 than G1and G2, with a significant difference (*P* = **0.02**; Fig. 5).

**Table 4:** Differential expression analysis of the studied miRNAs based on receptor status.

miRNA	G1; ER positivity N = 36	G2; HER2 positivity N = 28	G3; TNBC N = 5	*P*-value
miR-200a-3p	0.0017(0.0001–0.004)	0.019 (0.0026–0.19)	–	0.075
miR-205-5p	0.698 (0.22–1)	0.45 (0.25–1.27)	0.245 (0.159–1.159)	0.8
miR-124-3p	0.54(0.156–1.053)	0.53 (0.240- 0.990)	0.224 (0.10–1.24)	0.7
miR-15a-5p	1.6 (0.3 -3.9)	0.9 (0.5 – 2.2)	1.8 (0.4–5.1)	0.2
miR-155-5p	1.23 (0.24–2.5)	1.3 (0.5—3.1)	4.6 (2.8—19.2)	**0.02**

Data are expressed as median and interquartile range (IQR).

Bold indicated significant *P* value.

### Correlation coefficients between the studied miRNAs in BC patients

A Spearman Rank Correlation Coefficients have been performed to explore the associations between transcriptional levels of the following miRNAs (miR-200a-3p, miR-205-5p, miR-124-3p, miR-15a-5p, and miR-155-5p) and the results are recapitalized in Table 5. A strong miRNA correlation number was evaluated in patient samples. Except for miR-200a-3p, all the studied miRNAs (miR-205-5p, miR-124-3p, miR-15a-5p, miR-155-5p) showed a significant correlation in their expression profiles**.**

**Table 5:** Correlations between the relative expression levels among the interested miRNAs.

	miR-200a-3p	miR-205-5p	miR-124-3p	miR-15a-5p	miR-155-5p
miR-200a-3p	1.000	0.006	0.449	0.498	0.412
miR-205-5p		1.000	0.551**	0.483**	0.393*
miR-124-3p			1.000	0.390**	0.398*
miR-15a-5p				1.000	0.559**
miR-155-5p					1.000

*p ≤ 0.05, **p ≤ 0.01.

### Exploration of the utility of the miRNAs (200a-3p, 205-5p, and 124-3p) as potential biomarkers

**Figure 6:**
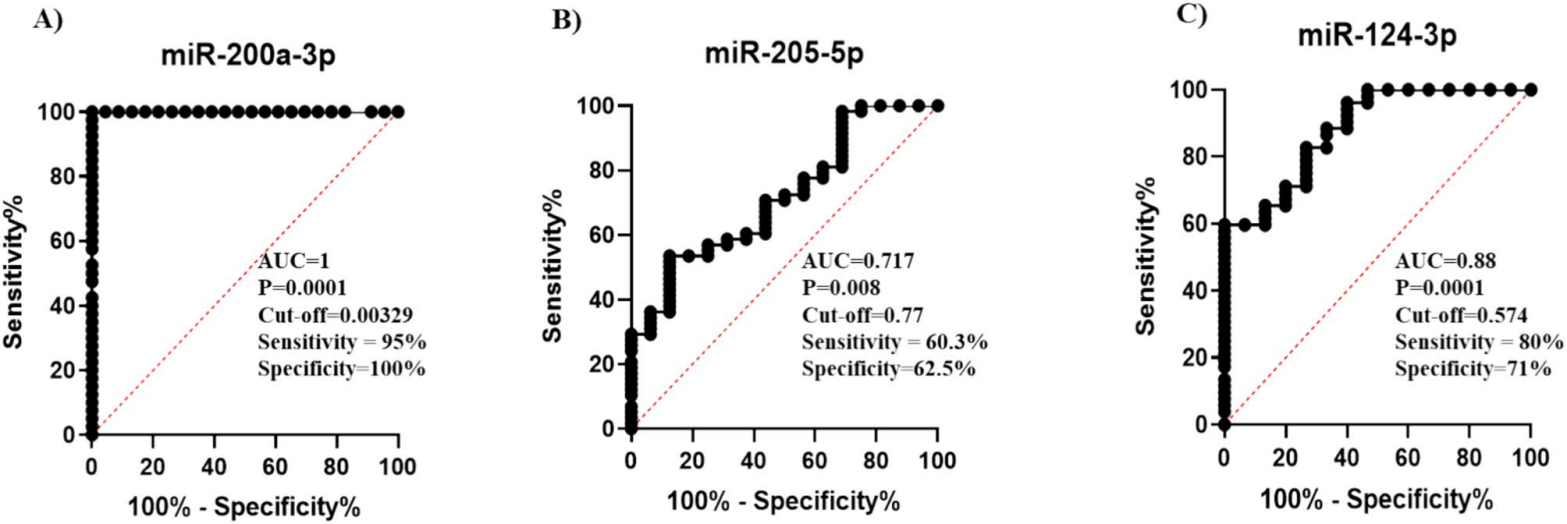
The area under the receiver operating characteristic (ROC) analysis was conducted for (**A**) miR-200a-3p, (**B**) miR-205-5p, and (**C**) miR-124-3p in BC patients relative to healthy individuals. The red line denotes no diagnostic value for the examined marker, whereas the other line represents the diagnostic value. A value closer to the uppermost corner indicates better diagnostic performance.

The potential ability of miR-200a-3p, miR-205-5p, and miR-124-3p was investigated to discriminate between breast cancer (BC) patients and healthy individuals by performing Receiver operating characteristic (ROC) analysis, as illustrated in Fig. 6. The results derived from the ROC curve analysis indicated that miR-200a-3p has excellent discriminatory performance, as evidenced by an estimated AUC of 1 (*P* = 0.0001), depicted in Fig. 6A. Furthermore, miR-205-5p and miR-124-3p exhibited good discriminatory performance, with estimated AUC values of 0.71 (*P* = 0.008) and 0.88 (*P* = 0.0001) respectively, as illustrated in (Fig. 6B, ).

## Discussion

Greater knowledge about the expression patterns of miRNAs- associated with cancer initiation, progression, angiogenesis, EMT, and metastasis- is crucial and will provide new opportunities for diagnostic and therapeutic approaches in BC. In this sense, miRNAs may serve as biomarkers for predicting systemic therapeutic response and prognosis in clinical applications. Novel therapies are halted mainly due to drug resistance and the lack of understanding of tumor cell signaling pathways^23^. In this regard, miRNAs can be used as targeted biomarkers for predicting disease progression and controlling chemotherapy resistance. The introduction of anti-oncomiR with the already established cancer treatment could lead to more successful treatment strategies^24,25^. unfortunately, limited studies are available on the role of miRNAs in post-transcriptional gene regulation in BC. The current research focused on the profiling of miRNAs involved in BC pathogenesis. MiRNAs exhibit dual roles as oncogenes or tumor suppressors, depending on the cancer type and the cellular context^26^. The miR-200a-3p belongs to the miR-200 family, the function of this family is mainly correlated with the suppression of EMT and tumor invasion. Consistent with the recent research, it was reported that the upregulation of miR-200a in Wilms tumor tissues can reduce survival rates and promote cancer cell apoptosis by inhibiting the Wnt/β-catenin signaling pathway^27^. Similarly, in the current data, the expression level of miR-200a-3p has shown a significant downregulation in BC patients compared to controls. Additionally, a slight decline has been observed in the late-stage group. Regarding the three hormonal classification groups, our results confirmed prior findings by others that there was no miR-200a-3p expression in TNBC^17^. The therapeutic effect of miR-200a-3p holds promising potential for several beneficial effects of adhesion junction stabilization, EMT decrement, and metastasis avoidance^28^. The expression of miR-200a-3p showed a progressive decline as the number of lymph node metastases increased^29^. Importantly and under certain conditions, the same transcript can induce EMT; its pleiotropic nature may highlight its versatility. Moreover, the oncologists recommended its usage to increase the efficacy of chemotherapy^30^. In BC, by suppressing *TGF-β2* expression, miR-200a-3p contributes to inhibit *TGF-β*-induced EMT^31^. Our in-silico analysis confirmed that the downregulation of the miR-200a-3p family leads to *TGF-β2* overexpression and influences the tumor's behavior.

It was reported that the miR-205-5p is defined as a tumor suppressor gene. Our data revealed the downregulation of the circulatory miR-205-5p in both early and late breast cancer patients compared to the healthy controls. This finding is supported by several studies that indicated the downregulation of miR-205-5p in tumors compared to normal tissue and lesser expression has been detected in metastatic tissue compared to control^32^. However, contradictory results reported that the upregulation of the circulatory miR-205-5p was correlated with tumor resistance to neoadjuvant chemotherapy in luminal A breast cancer^33^. On the other hand, recent research demonstrated that miR-205-5p is linked to luminal B tumor responsiveness to neoadjuvant chemotherapy.

Regarding the hormonal receptor status, our results reported that miR-205-5p is downregulated in TNBC relative to other types. This observation is consistent with the work of Radojicic et al.^34^ who stated that the miR-205-5p downregulation was linked to a poor prognosis within the TNBC subgroup. Moreover, our model-based predictions demonstrated the ability of miR-205 to inhibit the *VEGF* and *FGF2* expressions thus inducing apoptosis and enhancing response to chemotherapy^35^. Our in-silico analysis defined *VEGFA* and *FGF2* as efficacy biomarkers. Moreover, it was found experimentally that administering miR-205-5p and miR-141 in breast cancer tissues can reduce *VEGFA* metastasis and induce apoptosis^16,36^. It was found that the delivered mice with tumor xenografts from cells overexpressing miR-205-5p exhibited reduced tumor growth and increased sensitivity to doxorubicin^36^.

Concerning the miR-124-3p,it is defined as a tumor suppressor in many cancers including BC by inhibiting the translation of many oncogenic proteins^37^. In-vitro study on HepG2 cells showed that the miR-124-3p can repress *PIK3CA* expression by regulating the PI3K/Akt pathway and succeeding in inhibiting the cell tumorigenicity^38^. In our cohort, the decrement in miR-124-3p expression levels from control to both patients’ groups (early and late-stage groups) may contribute to tumorigenesis. Our results support the previous suggestions by Jia et al.^39^ for stimulating miR-124-3p expression to hinder BC cell proliferation and EMT. The study by Wang et al*.*^40^ defined the miR-124-3p along with the casitas B lineage lymphoma gene as a new regulatory axis that regulated the propagation and invasion of BC cells. Interestingly, our computational results recommended the inhibitory effect of miR-124-3p on *CDK6*, which functions as an inhibitor for both HER2-positive and TNBC^41^. Also, the current biomarker analysis suggested *STAT3* as an efficacy biomarker that is suppressed by miR-124-3p. It was reported that the induction of the miR-124 inhibits the STAT3 pathway and ameliorates the clinical outcome of radiotherapy^42^.

Regarding miR-15a-5p several evidence suggested that miR-15a-5p is downregulated in cancer cells. A recent study reported that the miR-15a-5p is downregulated in 80% of prostate cancer tissue compared to healthy tissue^43^. However, our data showed a slight increase in early and late-stage patients without significant differences. This finding is consistent with the work of Kodahl et al.^44^ who suggested the oncogenic property of miR-15a-5p. Regarding the hormonal receptor status, the miR-15a-5p expression level increases in the TNBC subgroup more than in other groups. It was found that the miR-15a-5p was significantly associated with the survival rate and aggressiveness of cancer cells^45^. Notably, it was concluded that the miR-15a-5p is more linked to prognosis than diagnosis. Moreover, the current in-silico study predicted the effect of this miRNA in inhibiting the expression of *CCNE1* and *VEGFA* which are linked to tumorigenesis and worse prognosis.

Recently, the introduction of Cyclin-dependent kinase4/6 (CDK4/6) inhibitors in the treatment strategy for breast cancer achieved great success, particularly in breast cancer patients with positive hormonal receptors. Interestingly, it was found that both miR-15-5p and miR-124-3p suppress the expression of *CDK6* which attracts attention toward these miRNAs to be used as therapeutic targets^41^.

The miR-155-5p has been defined as an oncogenic marker for different types of cancer including breast cancer that induces angiogenesis, tumor growth, and aggressiveness. However, there was inconsistency or even contradiction in the diagnostic accuracy of miR-155-5p in BC^46^. In our cohort, the circulatory miR-155-5p has shown a downregulation in the BC patients compared to the control. This finding is inconsistent with those who reported upregulation of miR-155-5p in BC tissue compared to those in normal tissue. However, a high tendency of elevation of this miRNA has been detected in the late-stage group^47^. Regarding hormonal receptor status, it was reported that the circulatory level of miR-155-5p was inversely associated with the expression of estrogen and progesterone receptors^48^. Similarly, our data revealed a significant elevation of miR-155-5p in the TNBC subgroup, these findings provide experimental support for using these miRNAs as a target for therapeutic intervention. Computational analysis showed that the miR-155-5p can activate genes with profound impact on cancer progression and tumor aggressiveness such as *CTNNB1* and inhibit DNA repair-related genes such as *ARID2* and *WEE1* as well as Transcription factor gene (*TCF4*)^49^. The activation of the Wnt signaling pathway is initiated through the binding of TCF4 to CTNNB1 and is associated with poor prognosis^50^. In an in-vivo study, it was found that the *CTNNB1* knockout activates the transcriptional activity of *TCF4* and suppresses the oncogenic pathway^50^. This elucidates the rationale behind the current simulated results showing the ability of miR-155-5p to inhibit *TCF4* and activate *CTNNB1*.

The correlation analysis of miRNA expression data revealed a positive correlation among miR-205-5p, miR-124-3p, miR-15a-5p, and miR-155-5p. However, miR-200a-3p did not exhibit significant correlations with the other miRNAs in breast cancer patients. These correlation results provide valuable insights into the interrelationships among different miRNAs in breast cancer patients. The diagnostic utility of the three significantly altered miRNAs has been assessed. The obtained data revealed that miRNA 200 a has excellent diagnostic accuracy while miR-205-5p and miR-124-3p had good diagnostic accuracy for identifying BC cases.

The current study was limited to a small sample size, particularly within the TNBC subgroup. The survival rate of BC patients is not estimated because this is not a longitudinal study and follow-up data is lacking. The Contradictory findings with some previous studies might be attributed to the differences in sample size, ethnic origin, variances in BC molecular and/or histopathological subtypes, and methodological differences.

## Conclusion

This study holds significant promise for developing noninvasive miRNA biomarkers with potential clinical applications. Three miRNA signatures (miR-200a-3p, miR-205-5p, and miR-124-3p) had potential diagnostic utility to discriminate between BC patients and control subjects. Further study is needed to evaluate the possibility of using the miR-155-5p as a therapeutic target in the TNBC subgroup. In the context of the cancer treatment approaches, the computational findings proposed that *EGFR*, *MAPK3*, *CCND1*, *FGF2*, *CHEK1*, *VEGFA*, *RELA*, and *ERBB3* have potential efficacy biomarkers for BC.

## Materials

### Samples collection

Serum samples were collected from 95 subjects (20 healthy controls and 75 patients). On a plain tube, two milliliters of whole blood were collected from each subject and the serum was separated from the blood and then preserved at − 80 °C till usage. According to the Helsinki Declaration (1975), this study was approved by the National Research Center's Ethical Review Board (NO., 09,410,224). All participants provided a written consent form for participation. All methods were carried out following relevant guidelines and regulations.

### In silico analyses for the studied miRNAs with BC

In silico analyses were conducted to elucidate the impact of significantly expressed miRNAs on breast cancer-related pathways. The QIAGEN’s Ingenuity Pathway Analysis (IPA) program (QIAGEN Inc., https://www.qiagenbioinformatics.com/products/ingenuity-pathway-analysis) was employed for target gene prediction, subcellular localization annotation and biomarker analysis. Krämer et al.^51^ described the algorithms used in QIAGEN IPA.

Supplementary Figure S1 Legend. Symbols of the candidate miRNAs (as referenced in the ingenuity software).

### miRNA isolation

Via the RNeasy Serum/Plasma Kit from (Qiagen, Germany) miRNAs were isolated from serum following the manufacturer's instructions. The quality of RNA samples was checked using a Thermo Scientific NanoDrop™ Spectrophotometer.

### miRNA reverse transcription

The Reverse-Transcription (RT) reaction was performed with a final volume of 20 μl, maintaining the reaction on ice. The reaction mix included 4 μl of 5 × miScript HiSpec Buffer (Qiagen, Germany), 2 μl of 10 × miScript Nucleics Mix (Qiagen, Germany), 2 μl of miScript RT Mix (Qiagen, Germany), 60 ng of Template RNA, and RNase-free water was added to bring it to the desired volume. After gentle pipetting and centrifugation, the mixture was incubated at 37 °C for 60 min, followed by 5 min at 95 °C to deactivate the mix of miScript RT. The cDNA volume was adjusted with RNase-free water to 110 μl and stored at − 20 °C till usage.

### Expression profile of the candidate miRNAs by qRT-PCR

The reagents required for qRT-PCR were mixed as follows: 2 μl of each candidate miRNA primer (10 μM; Qiagen, Germany), and 2 μl of cDNA were added to miScript PCR mix in a total volume of 20 μl. using miScript primer assays for miR-200a-3p (MS00009081), miR124-3p (MS00009085), miR-205-5p (MS00008185), miR-15a-5p (MS00008984), mir-155-5p (MS00003759). For all the candidate miRNAs, relative expressions were normalized to the housekeeping gene (HK) (SNORD61-1,531,894) that was quantified in a parallel reaction (purchased from Qiagen Hilden, Germany). The thermal profile was used as follows: initial incubation for 10 min at 95 °C (pre-activation), 40 cycles at 95 °C for 15 s, and 60 °C for 1 min. A rotor-gene real-time PCR system was used for the PCR run (Qiagen, Santa Clarita, CA).

### miRNA expression analysis

For miRNA expression analysis, the relative expression levels of miRNAs were calculated using the 2^−ΔΔCt^ method. Initially, the Ct values obtained from the amplification analysis of target miRNAs were normalized against the housekeeping gene SNORD61 (ΔCt). Subsequently, the ΔΔCt was determined by computing the difference between the normalized Ct values of target miRNAs in patients and the normalized Ct values of target miRNAs in controls. Samples with undetermined Ct values were excluded from the analysis.

For the miRNA expression analysis, the miRNA relative expression levels were determined using the 2^−ΔΔCt^ method described previously^52^. The studied miRNAs were normalized against the HK (SNORD61).

### Statistical analysis

The data were gathered and inputted into Statistical Package for Social Science (SPSS) version 26. Qualitative data results were expressed numerically and as percentages, for quantitative data with a parametric distribution, means were employed. In instances of non-parametric distribution, quantitative data were presented using the median with the interquartile range (IQR). The Spearman’s coefficient correlation was calculated, and the area under the receiver operating characteristic curve was assessed.

## Supplementary Information

Supplementary Information.

## Data Availability

The analytical tools, including "microRNA Target Filter" and "Biomarker Analysis" have been integrated and can be accessed through Ingenuity Pathway Analysis (IPA) at http://www.ingenuity.com.
