# Supplementary material for: Evaluation of tumorigenesis-related miRNAs in breast cancer in Egyptian women: a retrospective, exploratory analysis
Source: Sci Rep. 2024 Nov 29;14:29757. doi: 10.1038/s41598-024-68758-0 (PMC11607072; doi:10.1038/s41598-024-68758-0)
Supplement: Supplementary file 1 — Supplementary Information. [file 41598_2024_68758_MOESM1_ESM.pdf]

ID	Symbol
hsa-miR-124-3p	miR-124-3p (and other miRNAs w/seed AAGGCAC)
hsa-miR-200a-3p	miR-141-3p (and other miRNAs w/seed AACACUG)
hsa-miR-155-5p	miR-155-5p (miRNAs w/seed UAAUGCU)
hsa-miR-15a-5p	miR-16-5p (and other miRNAs w/seed AGCAGCA)
hsa-miR-205-5p	miR-205-5p (and other miRNAs w/seed CCUUCAU)

Figure S1. Illustrates the nomenclature of the selected miRNAs in the IPA database
